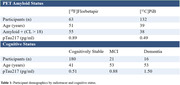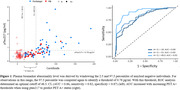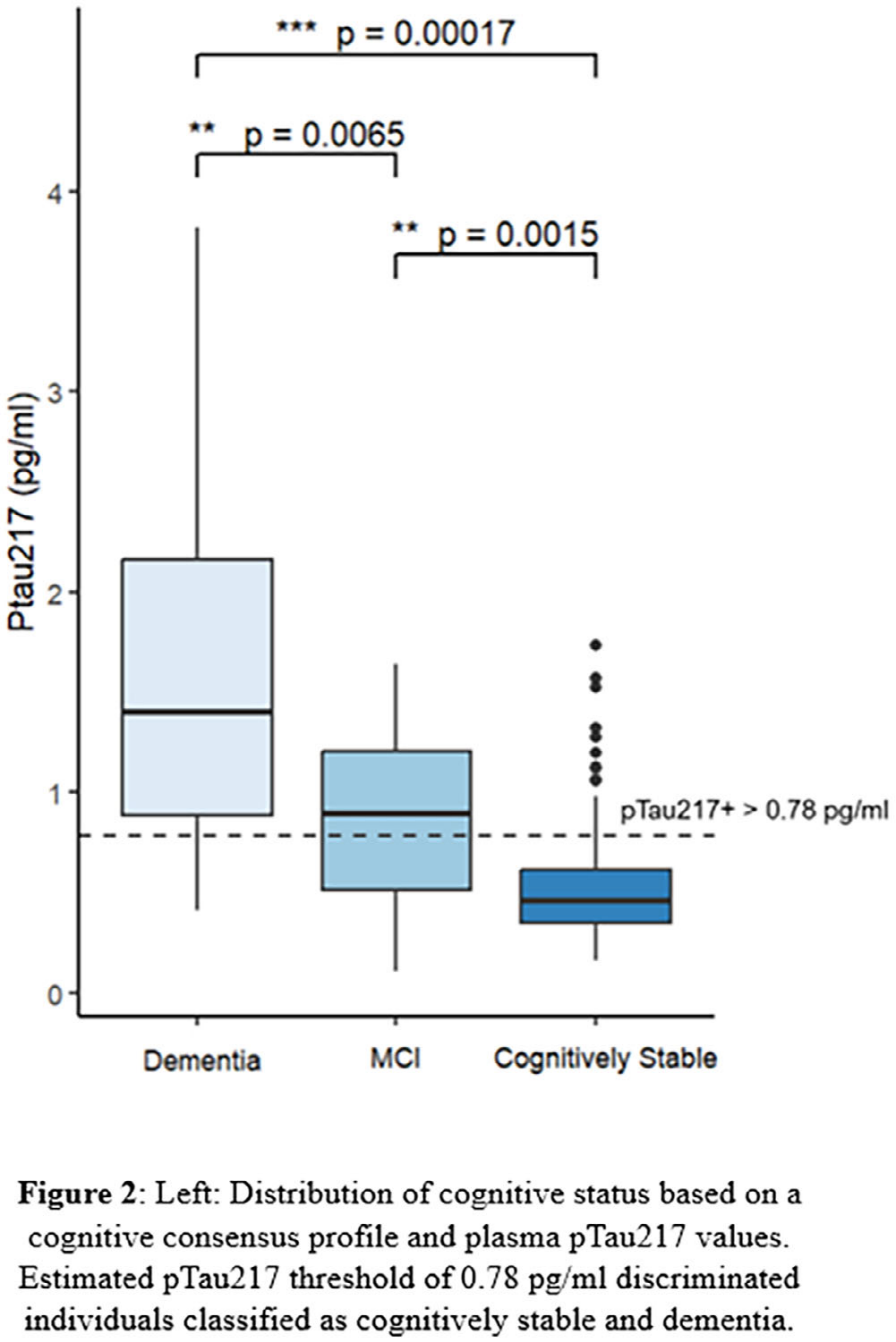# Associations of plasma phospho‐Tau217 with amyloid‐PET and cognitive status in adults with Down syndrome

**DOI:** 10.1002/alz.092599

**Published:** 2025-01-09

**Authors:** Brecca Bettcher, Shorena Janelidze, Max McLachlan, Matthew D Zammit, Andrew K McVea, Alexandra H DiFilippo, Tobey J. Betthauser, Charles M Laymon, Dana Tudorascu, Annie Cohen, Arun Garimella, Julie C Price, David B. Keator, Adam M. Brickman, Patrick J. Lao, William E Klunk, H. Diana Rosas, Shahid Zaman, Sigan L Hartley, Elizabeth Head, Mark Mapstone, Sharon J. Krinsky‐McHale, Sterling C. Johnson, Florence Lai, Beau Ances, Benjamin L Handen, Oskar Hansson, Bradley T. Christian

**Affiliations:** ^1^ Waisman Center, University of Wisconsin‐Madison, Madison, WI USA; ^2^ Lund University, Lund Sweden; ^3^ University of Wisconsin‐Madison School of Medicine and Public Health, Madison, WI USA; ^4^ University of Pittsburgh, Pittsburgh, PA USA; ^5^ Massachusetts General Hospital, Boston, MA USA; ^6^ University of California, Irvine, Irvine, CA USA; ^7^ Columbia University, New York, NY USA; ^8^ University of Cambridge, Cambridge UK; ^9^ New York State Institute for Basic Research in Developmental Disabilities, Staten Island, NY USA; ^10^ Washington University in St. Louis School of Medicine, St. Louis, MO USA

## Abstract

**Background:**

Blood‐based biomarkers able to detect atypical neuropathology could serve as a cost‐effective and noninvasive screening to include participants with Down syndrome (DS) in anti‐amyloid clinical trials. Accurately placing these novel biomarkers on the AD pathological cascade as proposed by the AT(N) framework informs relative disease progression of individuals. This work examines associations between plasma pTau217 accumulation, PET amyloid positivity, and cognitive status in adults with Down syndrome.

**Method:**

Participants were recruited from the Alzheimer’s Biomarker Consortium – Down Syndrome (ABC‐DS) study (Table 1). Amyloid positivity was determined by [^11^C]PiB or [^18^F]florbetapir (A+: Centiloids > 18) PET imaging from U01 cycle 1. Participants were classified as cognitively stable, mild cognitive impairment (MCI), or dementia (D). Plasma pTau217 concentration was measured using Lilly’s immunoassay for the Meso Scale Discovery platform. The threshold for ptau217 abnormality was derived using a subsample of amyloid negative participants. Youden’s Index (YI) was optimized to assess ptau217 sensitivity to PET A+ individuals. The time offset between PET amyloid positivity (18 CL) and ptau217 sensitivity was based on amyloid chronicity trajectories (Zammit et al, 2023). A second analysis was performed with respect to varying PET A+ thresholds.

**Result:**

Amyloid positive participants showed elevated pTau217 levels compared to amyloid negative participants (A+: 0.82 pg/ml, A‐: 0.44 pg/ml, p < 0.05). With a data derived pTau217 threshold of 0.78 pg/ml, optimization of YI identified an optimal cut point of 48.5 CL. In a ROC analysis, AUC increased with increasing PET A+ thresholds (Figure 1). A significant separation of pTau217 with cognitive status was observed (cognitively stable: 0.51 pg/ml, MCI: 0.88 pg/ml, D: 1.50 pg/ml) (Figure 2).

**Conclusion:**

Concordance was high between plasma pTau217 using Lilly’s immunoassay and amyloid PET status after reaching elevated amyloid levels of 48.5 CL or ∼6 years PET A+. This work suggests that elevated pTau217 levels follows Aβ neuropathology detected by PET and trends with clinical progression.